# CDK12 is a potential biomarker for diagnosis, prognosis and immunomodulation in pan-cancer

**DOI:** 10.1038/s41598-024-56831-7

**Published:** 2024-03-19

**Authors:** Ke-Qi Lu, Zuo-Lin Li, Qian Zhang, Qing Yin, Yi-Lin Zhang, Wei-Jie Ni, LiangYun-Zi Jiang, Wei He, Bin Wang

**Affiliations:** 1 Institute of Nephrology, Zhong Da Hospital, Southeast University School of Medicine, Nanjing, Jiangsu China; 2 Pediatric Surgery, The First Affiliated Hospital of Zhengzhou University, Zhengzhou, Henan China; 3 Department of Gastroenterology, Jiangsu Province Geriatric Institute, and Jiangsu Province Official Hospital, Geriatric Hospital of Nanjing Medical University, Nanjing, Jiangsu China

**Keywords:** CDK12, Pan-cancer, Diagnosis, Prognosis, Immunization, Gene mutation, Cancer, Computational biology and bioinformatics, Genetics, Immunology

## Abstract

Cell cycle-dependent protein kinase 12 (CDK12) plays a key role in a variety of carcinogenesis processes and represents a promising therapeutic target for cancer treatment. However, to date, there have been no systematic studies addressing its diagnostic, prognostic and immunological value across cancers. Here, we found that CDK12 was significantly upregulated in various types of cancers, and it expression increased with progression in ten cancer types, including breast cancer, cholangiocarcinoma and colon adenocarcinoma. Moreover, the ROC curves indicated that CDK12 showed diagnostic value in eight cancer types. High CDK12 expression was associated with poor prognosis in eight types of cancer, including low-grade glioma, mesothelioma, melanoma and pancreatic cancer. Furthermore, we conducted immunoassays to explore the exact mechanisms underlying CDK12-induced carcinogenesis, which revealed that increased expression of CDK12 allowed tumours to evade immune surveillance and upregulate immune checkpoint genes. Additionally, mutational studies have shown that amplification and missense mutations are the predominant mutational events affecting CDK12 across cancers. These findings establish CDK12 as a significant biological indicator of cancer diagnosis, prognosis, and immunotherapeutic targeting. Early surveillance and employment of CDK12 inhibitors, along with concomitant immunotherapy interventions, may enhance the clinical outcomes of cancer patients.

## Introduction

Cell cycle-dependent protein kinase 12 (CDK12) is a cell cycle-dependent protein kinase that functions as a transcription-related kinase. It forms a heterodimeric complex with Cyclin K, which phosphorylates the carboxyl-terminal domain of RNA polymerase II (RNA Pol II) to regulate gene expression. Convincing evidence has shown that CDK12 plays a pivotal role in various biological functions, such as transcription, pre-mRNA splicing, intronic polyadenylation (IPA) and translation. One noteworthy fact is that among the 20 members of the CDK family, only CDK12 is located on chromosome 17q12, which has been shown to consistently exhibit oncogenic features and genetic alterations across various types of tumors^1^. CDK12 is also the most frequently mutated of all CDKs, reaching up to 5%, and its genomic alterations have been identified in cancers, such as prostate cancer, breast cancer, and colorectal cancer^2^. The specific relationship between this and cancer has been strongly demonstrated in a considerable number of studies.

CDK12 has been found to drive and maintain the growth of cancer cells in a variety of tumours by regulating multiple signalling pathways, including the WNT/β-catenin pathway, MAPK pathway, NF-κB pathway, and DNA damage repair (DDR) pathway^3–7^. For instance, Liu et al. found that knocking out CDK12 in patient-derived tumour xenograft (PDX) models led to G2 cell cycle arrest and inhibited gastric cancer growth^6^. Moreover, growing evidence has demonstrated that CDK12 is a highly relevant gene in breast cancer development^3,5^. However, mutation analysis indicated that coamplification of the HER2 gene with its proximal 200 kb CDK12 was commonly observed in HER2-positive breast cancer, accounting for approximately 90% of cases^8,9^. Interestingly, this type of mutation has been shown to be associated with adverse prognosis and cancer recurrence^5^. In addition, CDK12 mutations were linked to rapid metastasis from primary lesions and progression of castration-resistant symptoms in metastatic prostate cancer^10^. Therefore, the inhibition of CDK12 expression can assist in cancer therapy.

Targeting CDK12 is currently a focus of precision therapy for cancer, and no specific CDK12 inhibitors have yet been clinically available. Bayles et al. discovered that E9, a dual inhibitor of CDK12 and CDK9, effectively treated metastatic osteosarcoma^11^. Additionally, coinhibitors of CDK12 and CDK13, such as THZ1, THZ531, and BSJ-01-175, derived from THZ531, all showed good efficacy in PDX mouse models of Ewing’s sarcoma^12,13^. Bajrami et al. utilized whole-genome high-throughput research to show that the absence of CDK12 was a decisive factor in increasing resistance to PARP1/2 inhibitors (PARPi) in cancer^14^. Using PDX models of triple-negative breast cancer (TNBC), one study found that dinaciclib, a pan-CDK inhibitor^15^, enhanced the anticancer efficacy of veliparib (PARPi) by inhibiting CDK12, and a phase I clinical trial of this combination for breast cancer is underway (NCT01434316)^16^. Moreover, when combined with a PARPi, the CDK12-specific inhibitor BSJ-4-116 exhibited strong antiproliferative activity in T-cell acute lymphoblastic leukemia^17^.

Therefore, a comprehensive and in-depth analysis of the correlation between CDK12 and cancer will provide new strategies for cancer management and treatment. In this study, we used a broader range of cancer types to investigate the significance of early monitoring of CDK12 expression in cancer diagnosis and prognosis. Additionally, we used immune infiltration analysis to explore the immunological mechanisms underlying the impact of CDK12 on cancer development. Our findings have potential implications for the development of new therapeutic approaches targeting CDK12 in cancer treatment.

## Results

### Differential expression of CDK12 between tumour and normal tissue samples

**Figure 1:**
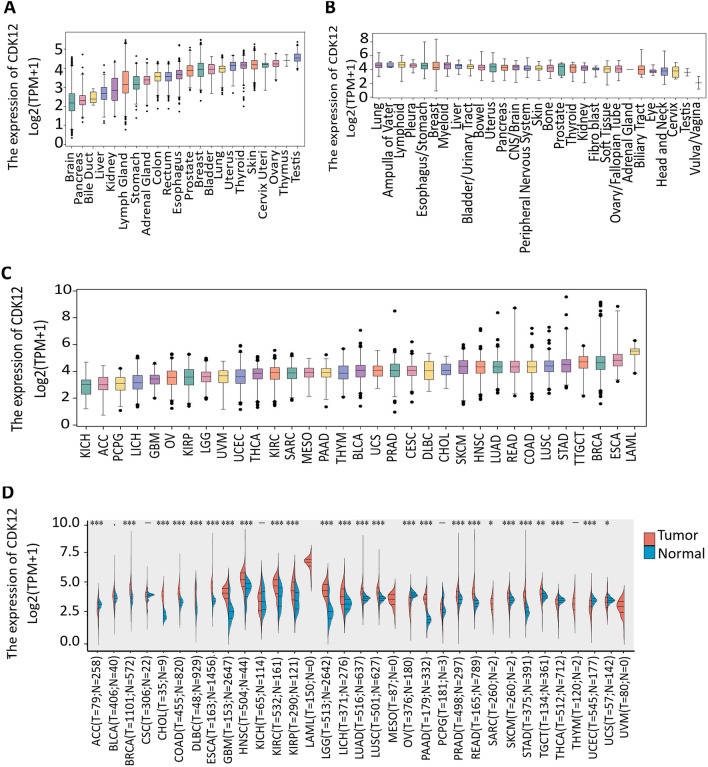
Differential expression of CDK12. (**A**) Expression of CDK12 in normal tissues. (**B**) Expression of CDK12 in cancer cell lines. (**C**) Expression of CDK12 in 33 types of cancer. (**D**) Comparison of CDK12 expression between tumor and normal samples. *p < 0.05, **p < 0.01, ***p < 0.001.*ns*not statistically significant.

**Figure 2:**
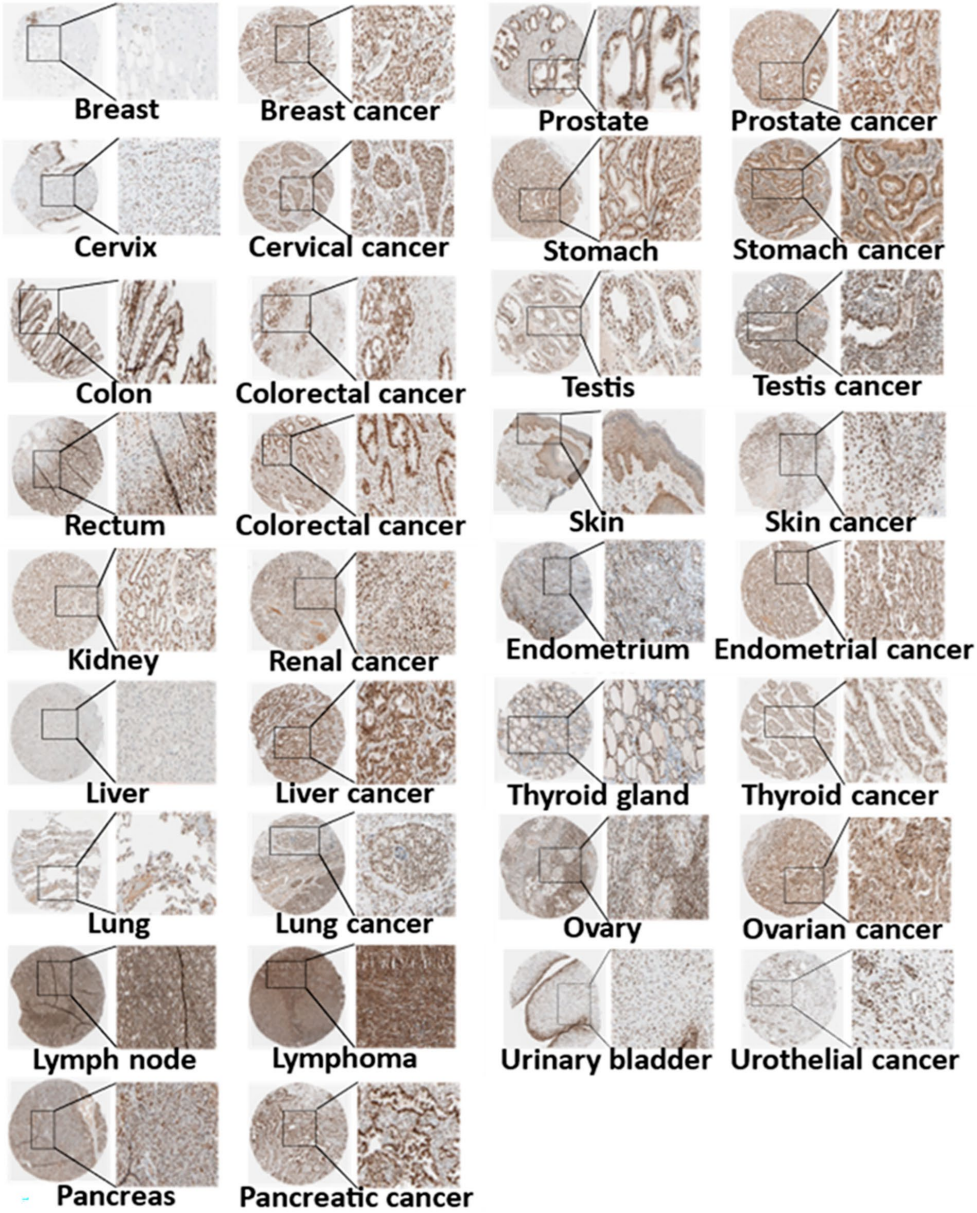
The protein expression of CDK12 in immunohistochemical images. Normal tissue (left) and tumor tissue (right).

The expression level of CDK12 in the GTEx and TCGA databases was sorted from low to high. The expression level of CDK12 was highest in the testes, and it was significantly higher in the testes than in other normal tissues. Furthermore, the expression level was lowest in the brain (Fig.1A). Moreover, the relative expression level of CDK12 in different cancer cell lines from the CCLE database was ranked from high to low. The expression level of CDK12 was generally upregulated in cancer cell lines of different tissue origins (Fig.1B), which was consistent with the expression of CDK12 in 33 types of tumours analysed by the TCGA database. LAML was the most highly expressed protein in cancer tissues, whereas KICH demonstrated the lowest expression levels (Fig.1C). Subsequently, the CDK12 expression levels in cancerous and paired normal samples from the GTEx and TCGA databases revealed significant differences from normal tissue in 25 cancers, excluding those without normal sample comparison (Fig.1D). In 20 types of cancer, CDK12 expression was upregulated compared to that in normal samples, whereas CDK12 was downregulated in ACC, OV, THCA, UCEC, and UCS compared with normal tissues. No significant difference in CDK12 levels was observed in CESC, KICH, PCPG, or THYM compared to nonmalignant tissues. These results indicated an upregulation of CDK12 in most cancer types, suggesting the potential key role of CDK12 in the diagnosis of cancer. Immunohistochemistry images from the Human Protein Atlas database indicate that CDK12 expression is higher in 12 types of cancers, including breast cancer, colorectal cancer, and liver cancer, compared to corresponding normal tissues (Table S1). In contrast, its expression is lower than normal tissues in three types of cancers: thyroid cancer, skin cancer, and endometrial cancer (Fig.2).

**Figure 3:**
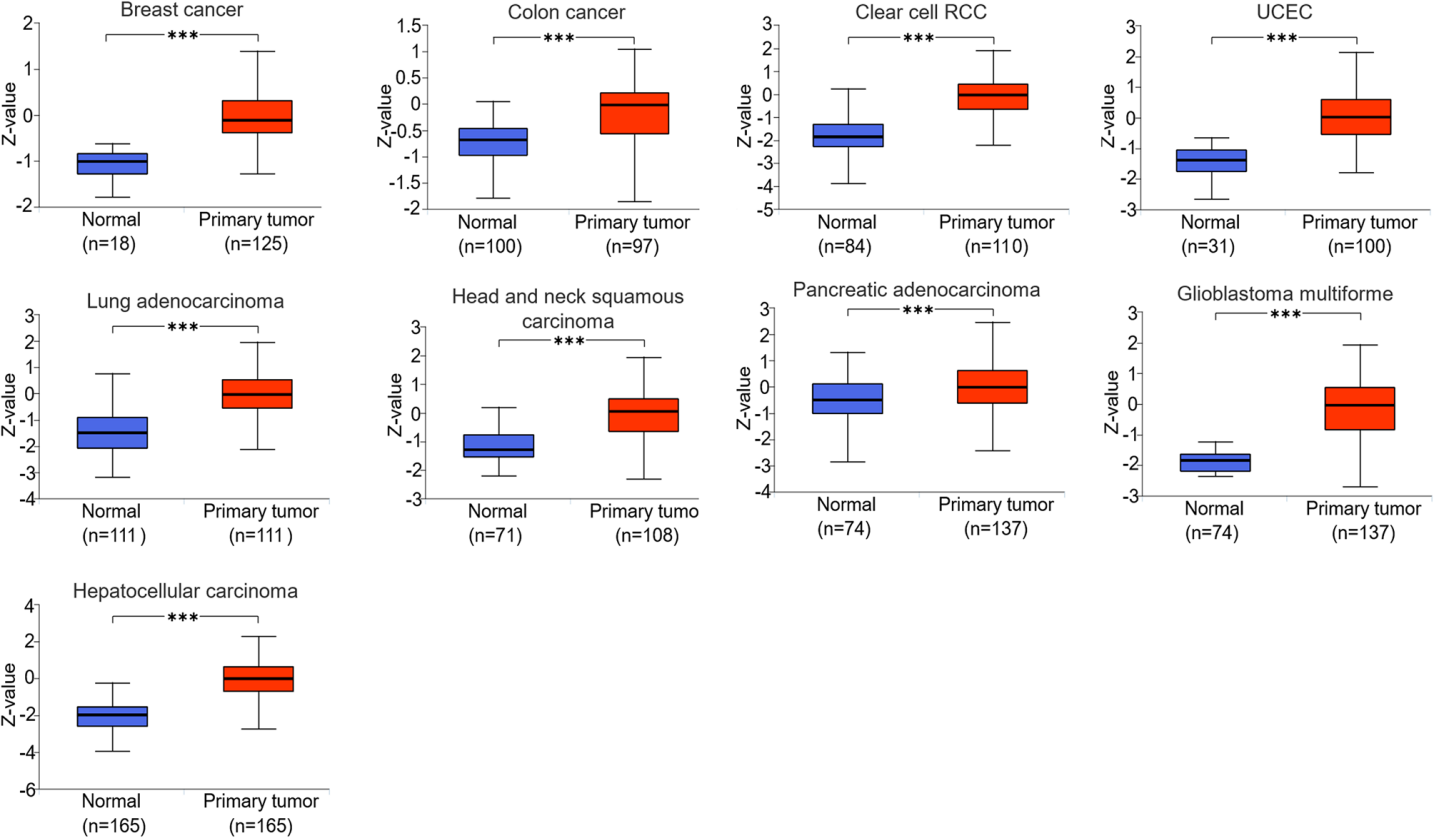
The protein expression level of CDK12 in cancers from CPTAC samples. *p < 0.05, **p < 0.01, ***p < 0.001.

In addition, according to the UALCAN database, we obtained the differences in protein expression of various cancers from the TCGA database, and the 9 cancer types with statistically significant differences were BRCA, COAD, KIRC, UCEC, LUAD, HNSC, PAAD, GBM and LIHC (Fig.3).

### Diagnostic value of CDK12 across cancers

**Figure 4:**
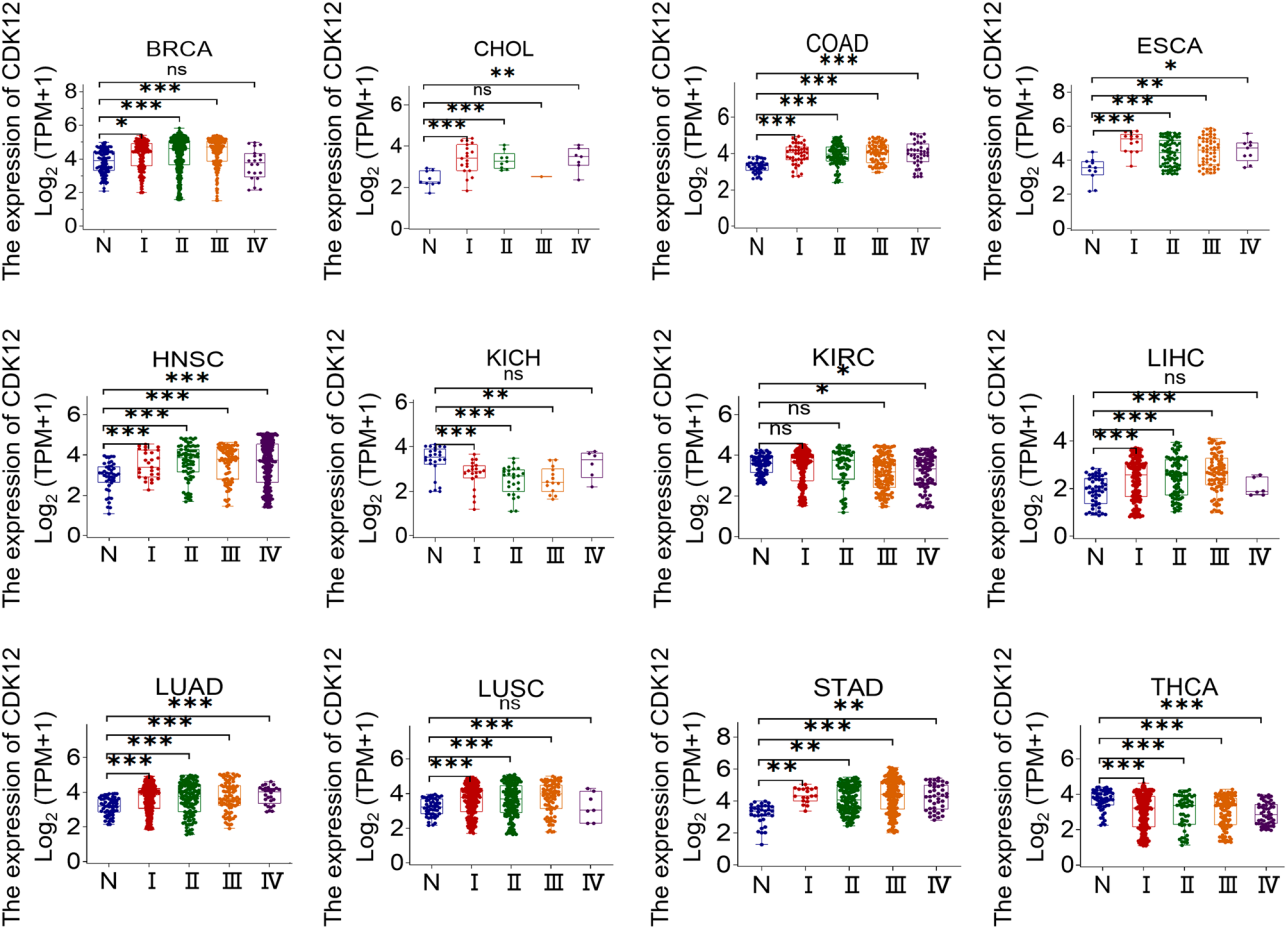
Association between CDK12 expression and tumor stage. *p < 0.05, **p < 0.01, ***p < 0.001. *ns*not statistically significant.

**Figure 5:**
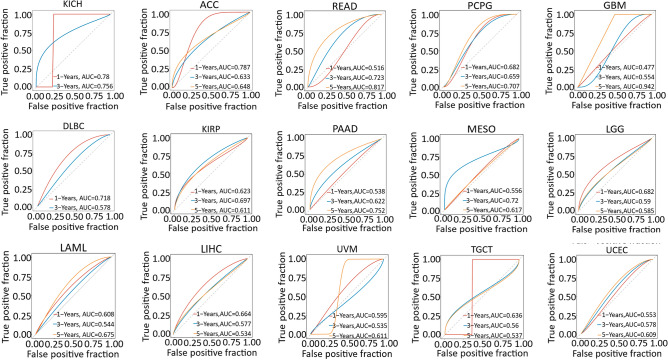
AUC of ROC curves verified the diagnosis performance of CDK12 in the TCGA cohort.

By visualizing the TNM staging of 12 types of cancer from the TCGA database examining the CDK12 expression levels (Fig.4), we found that 10 types of cancer had significantly higher CDK12 expression at advanced stages compared to earlier stages; these cancers included BRCA, CHOL, COAD, ESCA, HNSC, LIHC, LUAD, LUSC, READ and STAD. Conversely, CDK12 expression levels decreased with tumour progression in KICH and THCA. Overall, CDK12 may have certain clinical value in the early diagnosis of these tumours. Subsequent ROC curves were used to further study the diagnostic accuracy of CDK12 under 1-year, 3-year, and 5-year survival times of various types of tumours. Different AUC thresholds were considered to indicate high diagnostic accuracy (AUC: 1.0–0.9), relative diagnostic accuracy (AUC: 0.9–0.7), or low diagnostic accuracy (AUC: 0.7–0.5). As shown in Fig.5, the ROC analysis of the model had a relatively higher diagnostic accuracy of 0.942 under the 5-year survival time of GBM while being relatively lower in diagnosing the 7 types of cancers and lower still in diagnosing the 7 types of cancers.

### Prognostic significance of CDK12 across cancers

**Figure 6:**
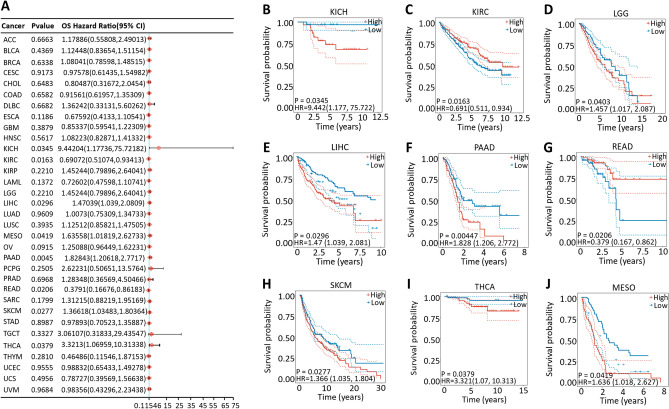
Association between CDK12 expression and overall survival (OS). (**A**) Forest plot of OS associations in 33 types of tumor. (**B–J**) Kaplan–Meier analysis of the association between CDK12 expression and OS.

**Figure 7:**
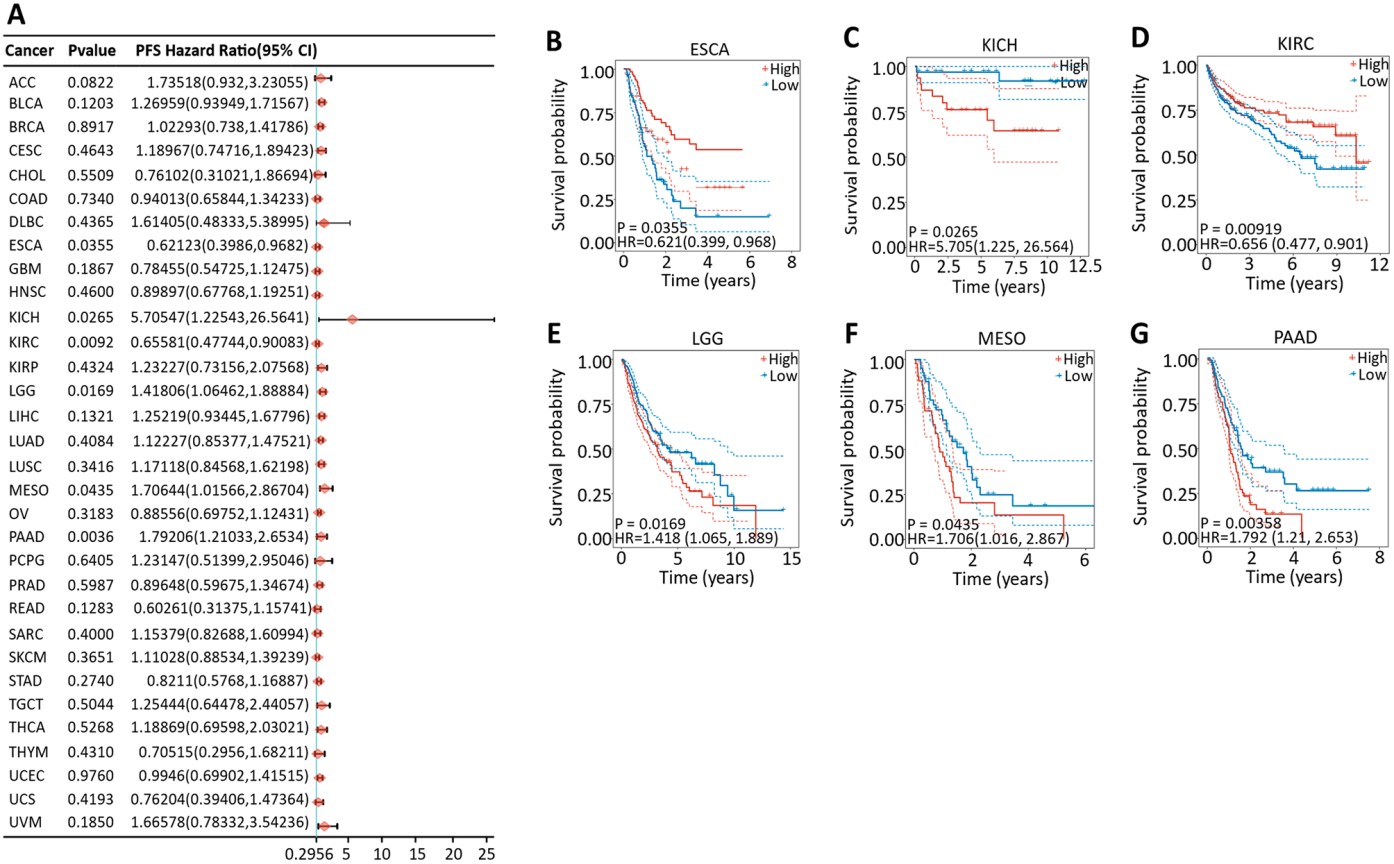
Association between CDK12 expression and progression-free survival (PFS). (**A**) Forest plot of PFS associations in 33 types of tumor. (**B–G**) Kaplan–Meier analysis of the association between CDK12 expression and PFS.

**Figure 8:**
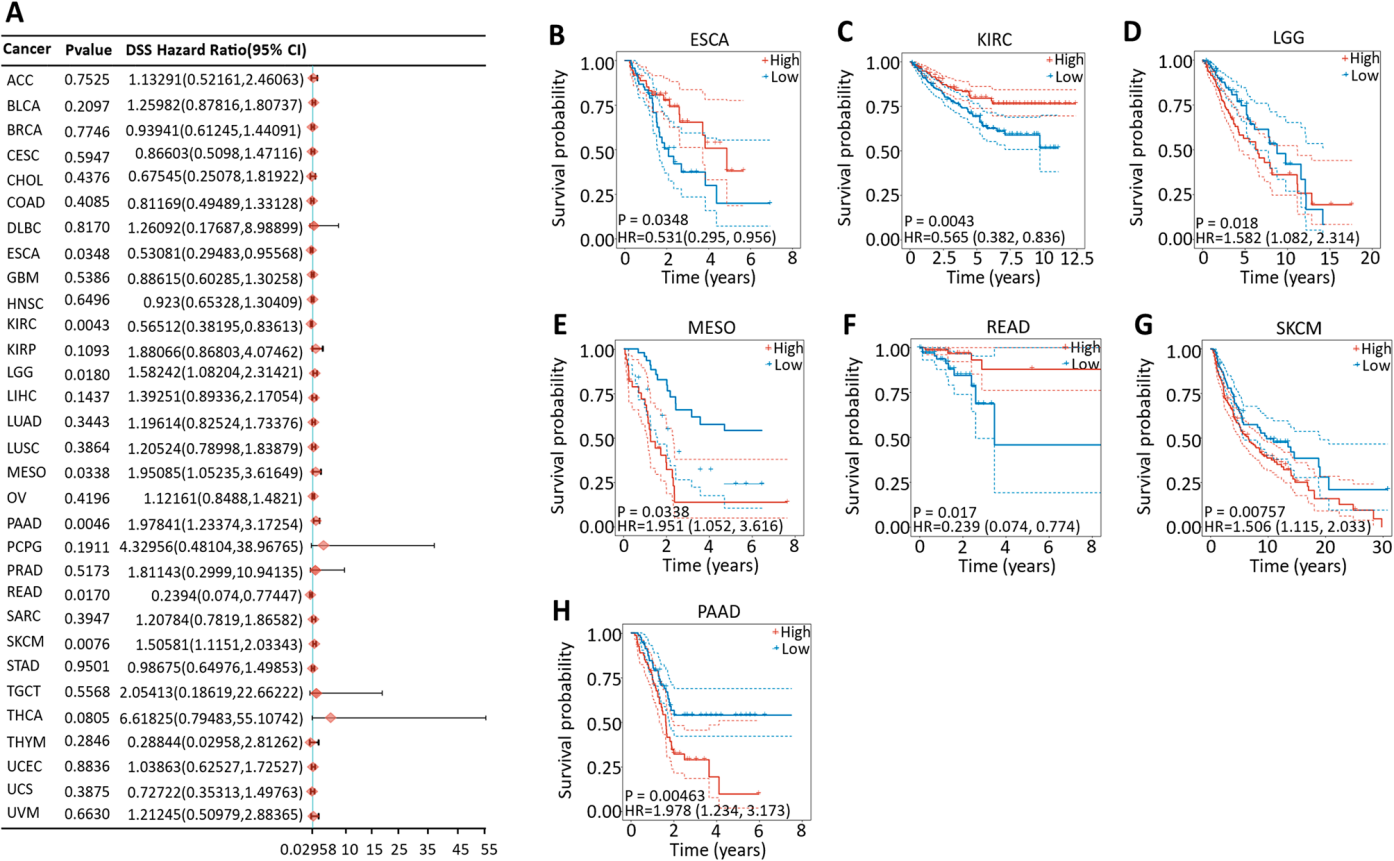
Association between CDK12 expression and disease-specific survival (DSS). (**A**) Forest plot of DSS associations in 33 types of tumor. (**B–H**) Kaplan–Meier analysis of the association between CDK12 expression and DSS.

Cox proportional hazard model analysis revealed that the CDK12 expression level was significantly correlated with the OS of KIRC, KICH, LGG, LIHC, MESO, READ, SKCM, THCA and PAAD (Fig.6A). However, CDK12 was a low-risk factor in KIRC and READ but a high-risk factor in other cancers, especially KICH, with an HR of 9.442 (Fig.6B–J). In addition, PFS data showed that the low expression of CDK12 in ESCA and KIRC was associated with poor prognosis, while the opposite was true in the other four cancers (Fig.7). The DSS analysis was consistent with the OS and PFS analysis results, which showed that the high expression of CDK12 was associated with poor prognosis in LGG, MESO, SKCM and PAAD, while the opposite was true in ESCA, KIRC and READ (Fig.8).

### Relationship between CDK12 expression and immune cell infiltration

**Figure 9:**
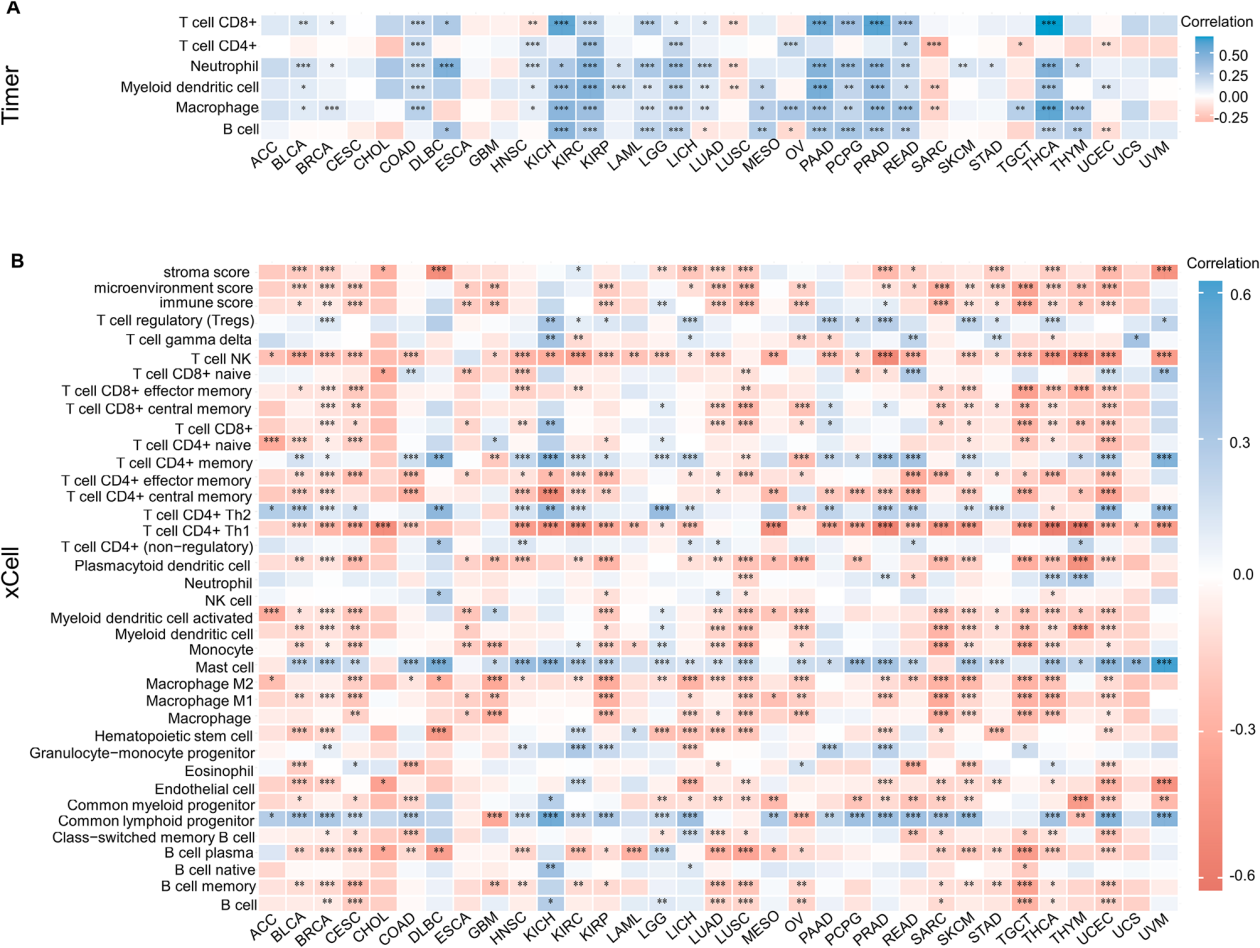
The CDK12 expression correlated with immune infiltration. (**A**) The CDK12 expression significantly correlated with the infiltration levels of various immune cells in the TIMER database. (**B**) The CDK12 expression significantly correlated with the infiltration levels of various immune cells based on CIBERSOR. *p < 0.05, **p < 0.01, ***p < 0.001.

We performed a pancancer analysis of the relationship between CDK12 expression and immune infiltration level based on the TIMER algorithm (Fig.9A). The expression of CDK12 was significantly correlated with the infiltration of immune cells. It was associated with B cells in 15 types of cancer, with CD4+T cells in 9 types of cancer, with CD8+T cells in 16 types of cancer, with macrophages in 19 types of cancer, with neutrophils in 20 types of cancer, and with dendritic cells in 18 types of cancer. Notably, most cancers exhibited positive correlations with these immune cell subtypes. Subsequently, the xCell new algorithm was employed to investigate the correlation between CDK12 expression and a wider range of immune cell subtypes (Fig.9B). We found that the expression of CDK12 was significantly inversely correlated with a majority of subtypes in BRCA, LUAD, LUSC, TGCT, OV, STAD, THCA and UCEC. In 33 cancer types, mast cells, CD4+Th2 T cells, Tregs, and common lymphoid progenitors were positively correlated with CDK12 expression. It should be noted that its expression was significantly negatively correlated with the stroma score, immune score and microenvironment score in most cancers.

**Figure 10:**
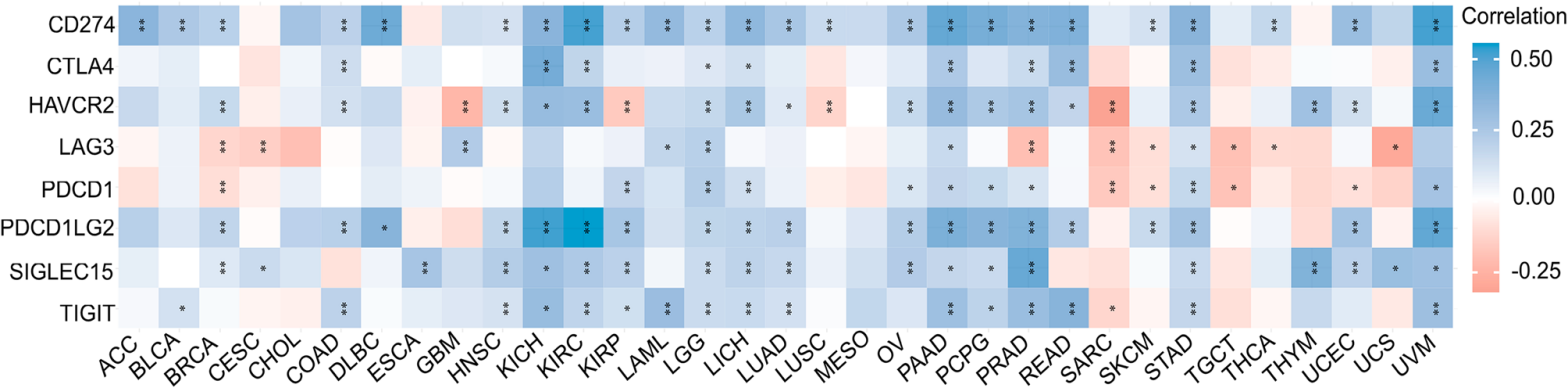
Correlation analyses of the CDK12 expression with immune checkpoint genes. *p < 0.05, **p < 0.01.

Convincing evidence indicates that tumours make use of immune checkpoints, such as PD-1, PD-L1, and CTLA-4, to evade the immune response^18^. To accurately estimate the association between CDK12 expression and the tumour microenvironment (TME) in a pancancer dataset, we then investigated the relationship between CDK12 expression and a variety of major types of immunomodulators (Fig.10). It is noteworthy that we observed an overall positive correlation between the expression of CDK12 and most immune checkpoint inhibitor and immune stimulatory molecules in LGG, LIHC, PAAD, STAD, and UVM. In contrast, the expression of CDK12 in SARC and TGCT was negatively correlated with most immune checkpoint inhibitor and immune stimulatory molecules. Overall, most immune checkpoint inhibitors and immune stimulatory molecules showed a positive correlation with the expression of CDK12.

### Correlation between CDK12 expression and TMB and MSI

**Figure 11:**
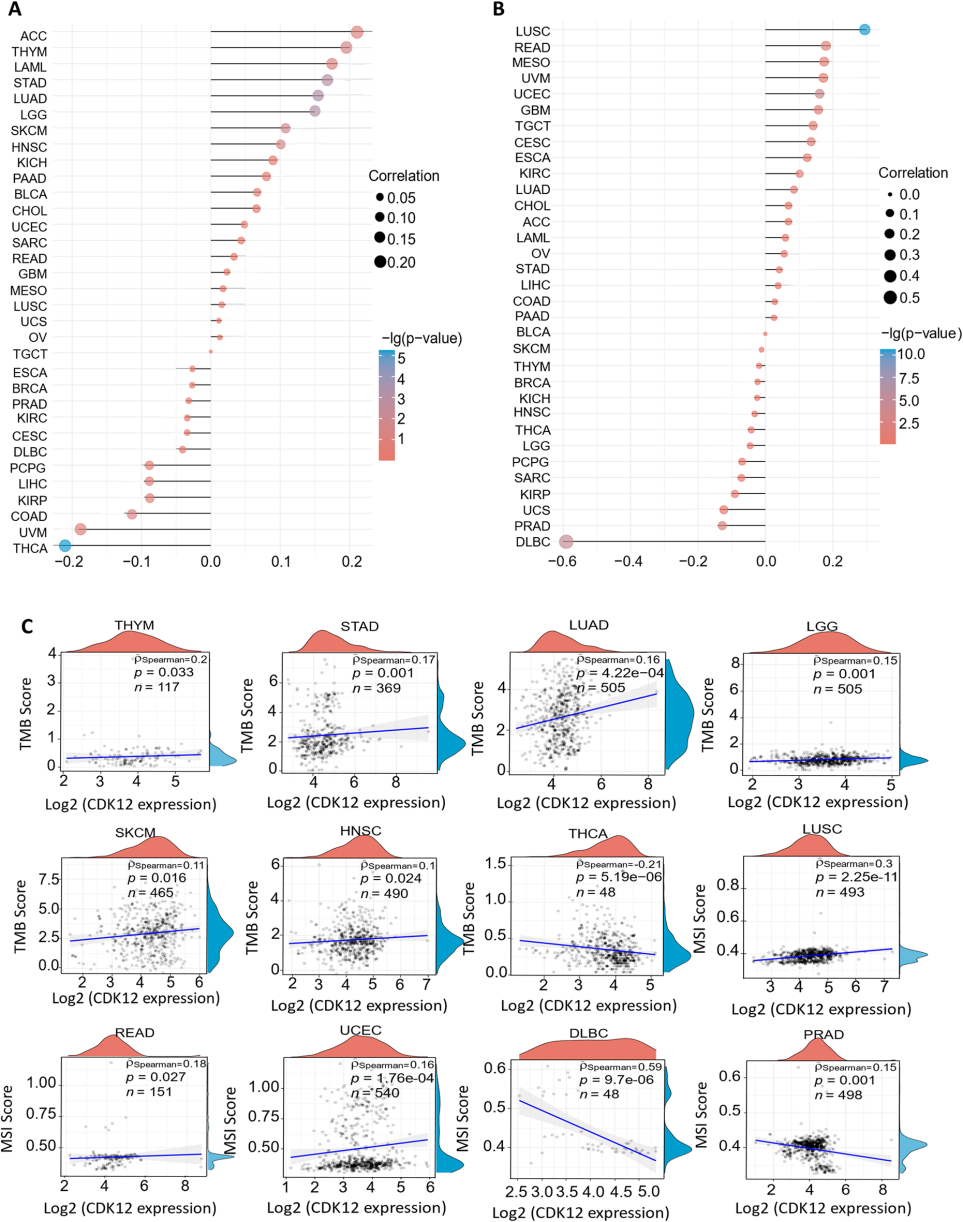
Spearman correlation analysis between the CDK12 gene expression and TMB and MSI. (**A**) A stick chart shows the relationship between the CDK12 gene expression and TMB in diverse tumors. The red curve represents the correlation coefficient, and the blue value represents the range. (**B**) A stick chart shows the association between the CDK12 gene expression and MSI in diverse tumors. (**C**) Relationship between the CDK12 gene expression and TMB or MSI in pan-cancer. Correlation analysis was performed using Spearman’s method.

TMB and MSI are two emerging biomarkers associated with the immune therapy response^19-21^. Through the analysis of CDK12 expression and its correlation with TMB (Fig.11A) and precise radar chart analysis (Fig.11C), it was observed that in seven types of tumours, namely, THYM, STAD, LUAD, LGG, SKCM, HNSC, and THCA, the expression level of CDK12 was significantly correlated with TMB. Additionally, it was negatively correlated with THCA. We investigated the correlation between CDK12 expression and MSI in 33 types of cancer (Fig.11B). There was a positive correlation in LUSC, READ, and UCEC and a negative correlation in DLBC and PRAD (Fig.11C).

### DNA methylation and genetic alteration analysis of CDK12

**Figure 12:**
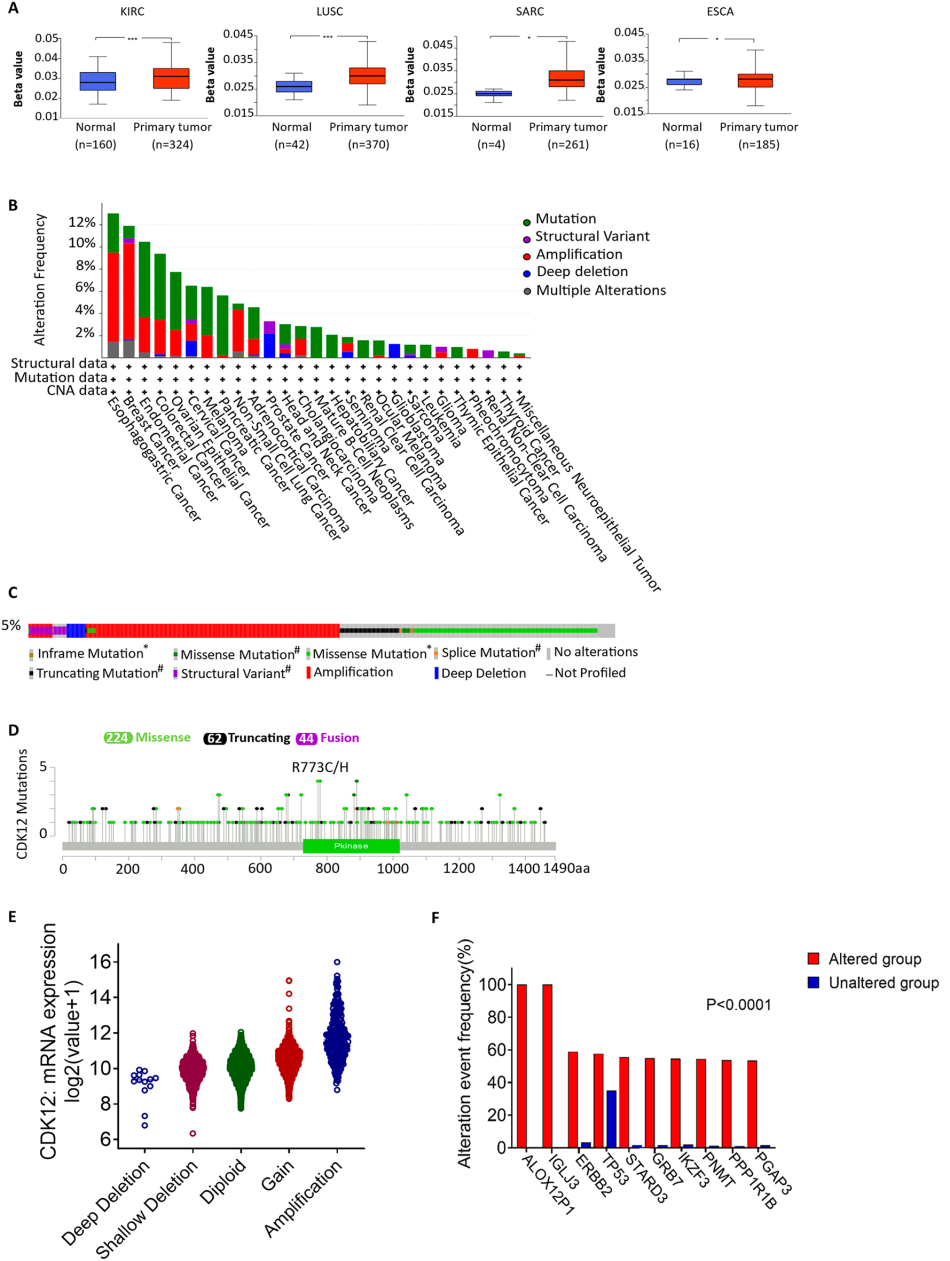
DNA methylation and mutation features of CDK12 in pan-cancer. (**A**) Promoter methylation level of CDK12 in pan-cancer. (**B**) Alteration frequency of CDK12. (**C**) OncoPrint visual summary of alterations in a query of CDK12 from cBioPortal. (**D**) The mutation types, number, and sites of the CDK12 genetic alterations. (**E**) The alteration types of CDK12 in pan-cancer. (**F**) The related genes alteration frequency in CDK12 altered group and unaltered group.

DNA methylation has a direct influence on the occurrence and development of cancer^22^. A study utilizing the UALCAN database revealed that there was a statistically significant difference in CDK12 promoter methylation levels in KIRC, LUSC, SARC, and ESCA compared to those in normal tissue (Fig.12A). Subsequently, pancancer alterations in CDK12 were examined using the cBioPortal platform. Of the types of cancers, oesophageal cancer had the highest mutation frequency of CDK12 at 12.94%; this was followed by breast cancer, bladder cancer, endometrial cancer, and colorectal cancer (Fig.12B). Among the various types of genetic alterations, amplifications and mutations were the most common types. Analysis of the common gene mutations of CDK12 also showed that amplifications, missense mutations and truncation mutations were the most common types, and the frequency of CDK12 somatic mutations in 10,967 samples was 5% (Fig.12C). Further research on the types, locations and case numbers of CDK12 gene modifications revealed that missense mutations were the main type of mutations (Fig.12D). For CDK12, the most common assumed copy number changes are amplifications, gain functions, and diploids (Fig.12E). After comparing CDK12 gene mutations and nonmutations, the ALOX12P1*, IGLJ3*, ERBB2, TP53, STARD3, GRB7, IKZF3, PNMT, PPP1R1B and PGAP3 genes were expressed at a relatively increased frequency in the mutation groups compared to the nonmutation groups (p < 0.0001), and these differences were statistically significant (Fig.12F).

### Interacting chemicals and CDK12 genes

**Figure 13:**
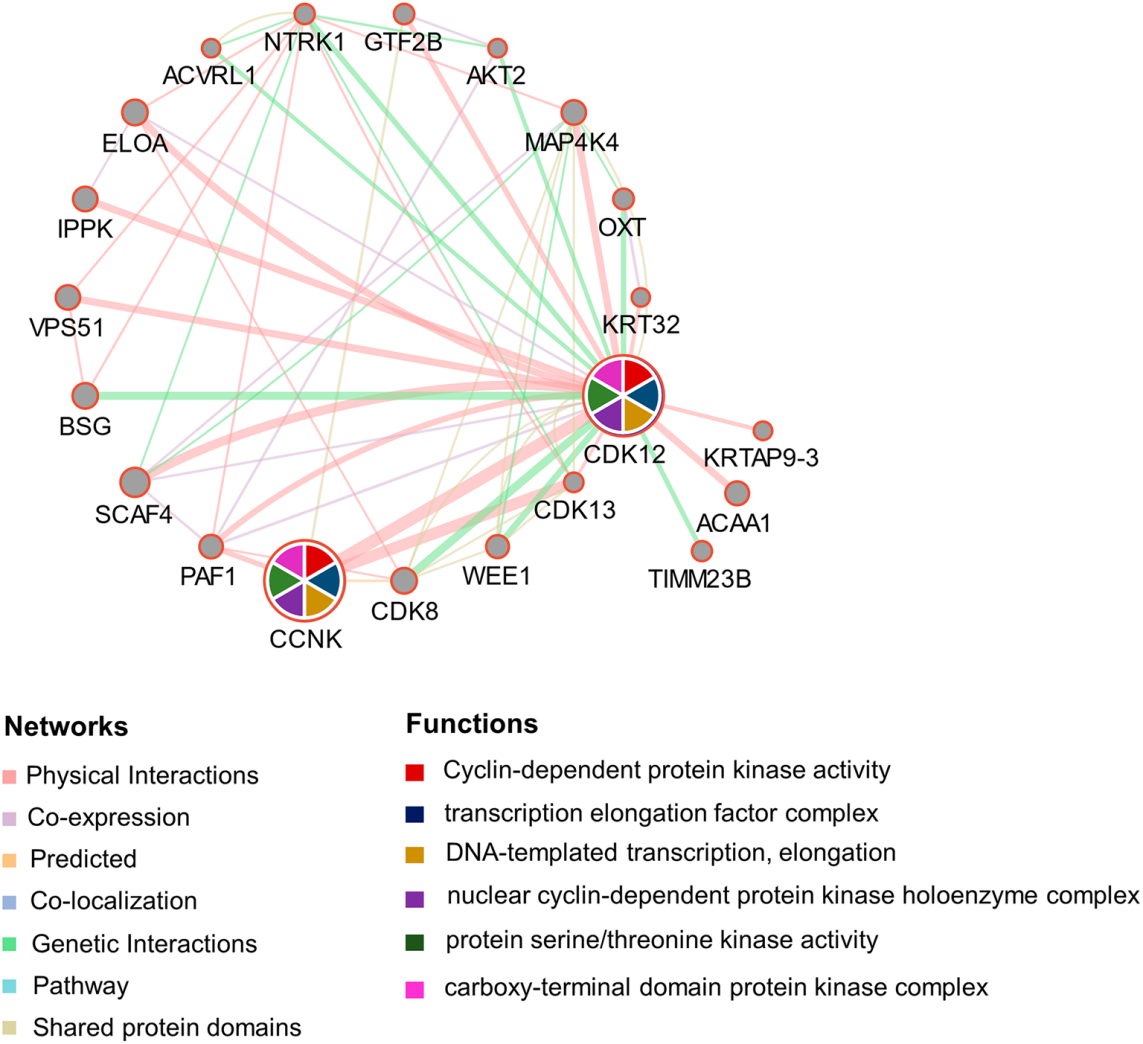
The gene–gene interaction network of CDK12 from GeneMANIA.

As shown in Table1, a total of 68 chemical substances related to CDK12 were integrated from the CTD database, among which 22 chemical substances could upregulate CDK12 and 35 substances had the opposite effect. Moreover, 11 substances were found to affect the expression of CDK12, but the specific function was still unclear. In addition, 20 genes with similar chemical associations with CDK12 were found, as shown in Table2. These genes included PINSR, GATAD2A, SF3B1, NIFK, LYSMD3 and PHY20L1, which are highly correlated with CDK12. A gene‒gene interaction network of CDK12 was constructed with GeneMANIA (Fig.13), which could intuitively identify the 20 most commonly altered genes closely related to CDK12. CCNK (Cyclin K) showed the most significant correlation with CDK12. Functional analysis revealed a significant correlation between CDK12 and its paralogues with the activities of CDKs, transcription elongation factor complexes and transcription elongation processes of template DNA.

**Table 1:** Interacting chemicals of CDK12 from CTD.

Chemical name	ID	Interaction actions	Chemical name	ID	Interaction actions
1,2-Dimethylhydrazine	DO19813	Affects expression	Glyphosate	C010974	Increases expression
2ʹ,3,3ʹ,4ʹ,5-Pentachloro-4-hydroxybiphenyl	C111118	Decreases expression	Haloperidol	D006220	Increases expression
2,3ʹ,4,4ʹ,5-Pentachlorobiphenyl	C070055	Increases expression	Hydralazine	D006830	Increases expression
2,4-Dinitrotoluene	C016403	Affects expression	Hydrogen peroxide	D006861	Increases expression
4-(5-Benzo(1,3)dioxol-5-yl-4-pyridin-2-yl-1H-imidazol-2-yl)benzamide	C459179	Decreases expression	ICG 001	C492448	Decreases expression
7,8-Dihydro-7,8-dihydroxybenzo(a)pyrene 9,10-oxide	D015123	Decreases expression	Magnetite Nanoparticles	D058185	Decreases expression
Acetaminophen	D000082	Increases expression	Methotrexate	D008727	Decreases expression
Aflatoxin B1	D016604	Increases expression	Methoxyacetic acid	C013598	Affects expression
Ammonium chloride	D000643	Affects expression	Methylmercuric chloride	C004925	Decreases expression
Antigens, polyomavirus transforming	D000952	Decreases expression	Methyl Methanesulfonate	D008741	Increases expression
Benzo(a)pyrene	D001564	Decreases expression	Mono-(2-ethylhexyl)-phthalate	C016599	Decreases expression
Bisphenol A	C006780	Decreases expression	*N*-Methyl-3,4-methylene-dioxyamphetamine	D018817	Decreases expression
Butyraldehyde	C018475	Decreases expression	Particulate matter	D052638	Decreases expression
Carbamazepine	D002220	Affects expression	Perfluoro-*n*-nonanoic acid	C101816	Decreases expression
Carbon tetrachloride	D002251	Increases expression	Perfluorooctanesulfona-mide	C063900	Decreases expression
Chlordecone	D007631	Increases expression	Perfluorooctane sulfonic acid	C076994	Decreases expression
Chloroprene	D002737	Increases expression	Perfluorooctanoic acid	C023036	Decreases expression
Chlorpyrifos	D004390	Increases expression	Phenobarbital	D010634	Affects expression
Cisplatin	D002945	Affects expression	Pirinixic acid	C006253	Affects expression
Copper	D003300	Affects expression	Silicon dioxide	D012822	Increases expression
Coumarin	C030123	Decreases expression	Sodium arsenate	C009277	Increases expression
Cyclosporine	D016572	Decreases expression	Sodium arsenite	C017947	Increases expression
Dicrotophos	C000944	Increases expression	Soman	D012999	Increases expression
Diisobutyl phthalate	C025605	Decreases expression	Succimer	D004113	Decreases expression
Dinitrochlorobenzene	D004137	Decreases expression	Tetrachlorodibenzodioxin	D013749	Affects expression
Diphenylcyclopropenone	C029402	Decreases expression	Thioacetamide	D013853	Increases expression
Dorsomorphin	C516138	Decreases expression	Trichloroethylene	D014241	Decreases expression
Doxorubicin	D004317	Decreases expression	Tris(1,3-dichloro-2-propyl)phosphate	C016805	Decreases expression
Entinostat	C118739	Decreases expression	Troglitazone	D000077288	Decreases expression
Ethinyl estradiol	D004997	Increases expression	Tungsten	D014414	Decreases expression
Ethylene dichloride	C024565	Increases expression	Urethane	D014520	Increases expression
Fipronil	C082360	Decreases expression	Valproic acid	D014635	Affects expression
Folic acid	D005492	Decreases expression	Vanadates	D014638	Decreases expression
Gentamicins	D005839	Increases expression	Vinclozolin	C025643	Decreases expression

**Table 2:** Relationship of CDK12 with genes via chemical interaction, based on the CTD database.

Gene	Similarity index	Common interacting chemicals
PINSR	0.34127	43
GATAD2A	0.33333	37
SF3B1	0.32203	38
NIFK	0.31933	38
LYSMD3	0.31	31
PHF20L1	0.30081	37
WDR33	0.29565	34
NKTR	0.29412	35
SENP7	0.29358	32
ZCCHC7	0.29032	36
CDC5L	0.28829	32
IFT57	0.28829	32
MGA	0.28723	27
USP37	0.28723	27
CSTF3	0.28689	35
LPGAT1	0.28682	37
INTS12	0.28571	26
LRRC20	0.28571	32
MLLT3	0.28571	34
PPP1R13L	0.28571	34
RNF44	0.28571	30
TAF1D	0.28571	38

## Discussion

To the best of our knowledge, there is no systematic research on the diagnostic, prognostic, and immunological value of CDK12 in cancer. In this study, we found that early monitoring of CDK12 expression could assist in diagnosing multiple types of cancer and predicting poor prognosis. Moreover, we elucidated the mechanisms by which CDK12 helps tumours evade immune surveillance and proposed a new approach for developing CDK12-specific inhibitors.

Previous studies have indicated that CDK12 is involved in promoting the development of various cancers, including THCA, BRCA, LIHC, READ, and STAD^5–9,22,23^. For instance, in breast cancer, it has been demonstrated that CDK12 activates the Erb-PI3-AKT or WNT/β-catenin signalling pathways by phosphorylating RNA Pol II, thereby contributing to cancer cell initiation, invasion, and sustained self-renewal^3^. Gastric cancer progression is highly correlated with the MAPK signalling pathway, and CDK12 can affect this pathway by phosphorylating the PAK2 gene, leading to the metastasis of gastric cancer^6^. In addition, the research team discovered that CDK12 is widely expressed in hepatocellular carcinoma (HCC) cell lines, confirming its critical role in HCC cell proliferation^24^. In our study, we identified new cancer types that were strongly related to CDK12. Moreover, high expression levels of CDK12 were significantly associated with cancer cell invasion and metastasis, as well as poor prognosis in multiple types of cancer. Combining the above findings, we conducted the first diagnostic accuracy study of CDK12 across cancers. Although the AUC value did not reach significance, CDK12 still showed early diagnostic value in multiple cancer types. Monitoring CDK12 expression and intervening in early-stage cancer has significant implications for diagnosing cancers and improving patient outcomes.

The TME and immunotherapy have emerged as prominent areas of clinical inquiry^25^. We have found that in most cancers, high CDK12 expression leads to the proliferation of various immune cells, such as tumour-associated macrophages (TAMs), neutrophils, and helper T cells (Th). TAMs can express chemokines and cooperate with other immune-suppressive cells to recruit and amplify Tregs. Tregs are considered a major obstacle to cancer immunotherapy. Immune cells, such as neutrophils, Th cells, and TAMs, can secrete immunosuppressive factors, and an increase in these cells can help cancer cells escape immune surveillance^26^. In a study conducted on prostate cancer, CDK12-mutated tumours showed higher T-cell infiltration and more expansion of cloned T cells relative to other prostate cancer genomic subtypes. Additionally, the expression levels of certain chemokines and their receptors were increased, which is consistent with the results of our study^27^. A higher stromal score, immune score and microenvironment score, which is the typical immune scoring system in the TME, represents a better prognosis for cancer. Conversely, low microenvironment and stromal scores were associated with poor prognosis in LIHC and LUAD^28,29^. The prognostic value of the immune score was reflected in situ and in metastatic colorectal cancer tissue^30^. In our study, CDK12 was negatively correlated with all three scores. These findings suggest that the high expression of CDK12 may contribute to cancer initiation and progression through immune mechanisms, laying the theoretical foundation for CDK12 inhibitors in combination with immunotherapy.

Emerging evidence has revealed that a combination of CDK12 inhibitors and PD-1 antibodies improves the prognosis of breast cancer patients^31,32^. Antonarakis et al. found that progressive prostate cancer with CDK12 somatic loss-of-function mutations responded well to PD-1 inhibitors^27^. We have uncovered that high expression levels of CDK12 lead to elevations in the expression levels of common immune checkpoint genes, such as PDCD1 and CTLA-4, providing evidence for the combination of CDK12 inhibitors and immune therapy. Since PD-1 inhibitor monotherapy is only effective in 20–40% of patients, monitoring the levels of TMB and MSI prior to initiating immune checkpoint inhibitor (ICI) therapy can improve the efficiency of immune therapy^33–35^. To the best of our knowledge, this is the first pancancer study to focus on the relationship between CDK12 and TMB/MSI. Several studies have demonstrated that patients with high TMB and MSI tend to derive greater benefit from immunotherapy. In most cancers, such as lung cancer, liver cancer, and colorectal cancer, patients with TMB-H can obtain long-term survival benefits from ICI therapy compared to those in the low TMB group^36,37^. MSI is due to aberrant function of the DNA mismatch repair system. MSI-H colon cancer patients displayed superior PD-1 inhibitor response, as well as longer median PFS and OS^38–40^. We identified a negative correlation between CDK12 and TMB/MSI in THCA, PRAD and DLBC. Adjuvant application of CDK12 inhibitors alongside ICI treatment may yield improved prognosis for patients with these malignancies.

The development of specific inhibitors for CDK12 has been considerably impeded by the high sequence homology shared with CDK13. Our study summarized numerous chemical compounds that can modulate the expression of CDK12, of which clinically used drugs, such as cyclosporine, quercetin, and entinostat, have exhibited inhibitory effects on CDK12. Initiating the development of CDK12 inhibitors using clinically established drugs could represent a promising strategy. Liu et al. identified that procaterol, a common clinical drug, can significantly restrict CDK12 kinase activity and inhibit the proliferation of human gastric cancer cells^6^. Mechanistically, CDK12 inhibitors primarily exert their anticancer effects by enhancing the anti-aggregation effect in cancer cells, reducing Pol II CTD Ser2 phosphorylation to inhibit DDR gene expression, and synergizing with poly (ADP-ribose) PARPi^5,14,16^. We screened for genes with more chemical cross-linking to CDK12. Among them, SF3B1 is a crucial splicing factor formed by DDR proteins. It promotes efficient mRNA splicing and is correlated with several types of cancer, including myelodysplastic syndromes and breast cancer^41,42^. CDK12 is a key gene regulating mRNA splicing and the DDR pathway. Further investigation is encouraged regarding the association between SF3B1 and CDK12. Antibodies related to these genes also provide new perspectives into the development of CDK12 inhibitors.

Given that our research heavily relies on bioinformatics techniques and utilizes public databases, it is subject to several limitations. Primarily, our dataset lacks experimental and clinical validation, as it is entirely sourced from public databases. Additionally, while CDK12 is highly correlated with the tumour immune microenvironment and response to immunotherapy, its underlying mechanisms remain largely unexplored. Hence, more in vitro and in vivo studies are required to further investigate their relationship and to corroborate our findings from clinical practice.

In summary, our study systematically analysed the diagnostic, prognostic, and immunological relevance of CDK12 across cancers, and it may serve as an ideal biological marker for early cancer diagnosis and the prediction of patient prognosis. Furthermore, the use of CDK12 inhibitor adjuvant immunotherapy, which can increase the cure rate and improve the prognosis of patients, needs to be put on the agenda as soon as possible.

## Materials and methods

### The expression pattern of CDK12 in human pancancer tissues

Gene expression data of 31 types of normal tissues from the GTEx database (https://commonfund.nih.gov/GTEx), with the addition of mRNA expression profiles and clinical data of 33 types of cancer and corresponding normal samples from the TCGA database (https://www.cancer.gov/aboutnci/organization/ccg/research/structural-genomics/tcga) downloaded through the Genomic Data Commons (GDC) portal, were collected. A total of 26,801 samples were involved. CDK12 expression was analysed between different cancer types and paired normal samples. Moreover, we extracted the data of 37 human tissue cancer cell lines from the CCLE database (https://sites.broadinstitute.org/ccle), involving 1409 samples. Patients with a total of 33 types of cancer were enrolled in RNA sequencing and clinical monitoring data; these cancers included adrenocortical carcinoma (ACC), bladder urothelial carcinoma (BLCA), breast invasive carcinoma (BRCA), cervical squamous cell carcinoma (CESC), cholangiocarcinoma (CHOL), colon adenocarcinoma (COAD), diffuse large B-cell lymphoma (DLBC), oesophageal carcinoma (ESCA), glioblastoma multiforme (GBM), low-grade glioma (LGG), head and neck squamous cell carcinoma (HNSC), kidney chromophobe (KICH), kidney renal clear cell carcinoma (KIRC), kidney renal papillary cell carcinoma (KIRP), acute myeloid leukaemia (LAML), liver hepatocellular carcinoma (LIHC), lung adenocarcinoma (LUAD), lung squamous cell carcinoma (LUSC), mesothelioma (MESO), ovarian serous cystadenocarcinoma (OV), pancreatic adenocarcinoma (PAAD), pheochromocytoma and paraganglioma (PCPG), prostate adenocarcinoma (PRAD), rectum adenocarcinoma (READ), sarcoma (SARC), skin cutaneous melanoma (SKCM), stomach adenocarcinoma (STAD), testicular germ cell tumour (TGCT), thyroid carcinoma (THCA), thymoma (THYM), uterine corpus endometrial carcinoma (UCEC), uterine carcinosarcoma (UCS) and uveal melanoma (UVM). All the data were normalized and transformed using log2 and tested with a t test, with a p value < 0.05 as the standard of abnormality. Data analysis was performed using R software (version 4.0.3, https://www.R-project.org), and a boxplot was plotted using the “ggplot2” R package.

### Immunohistochemical staining of CDK12

The Human Protein Atlas (HPA, https://www.proteinatlas.org/) database contains a human proteome map of protein expression and distribution in human tissue and cells. To evaluate the difference in the protein level of CDK12, immunohistochemistry images of 17 types of tumours, including breast cancer, cervical cancer, colorectal cancer, endometrial cancer, renal cancer, liver cancer, lung cancer, lymphoma, ovarian cancer, pancreatic cancer, prostate cancer, skin cancer, gastric cancer, testicular cancer, and thyroid cancer, and their corresponding normal tissues from the HPA database were downloaded and analysed using Image J software (Supplementary TableS1).

### Correlation between CDK12 expression and DNA methylation

The UALCAN database (http://ualcan.path.uab.edu/analysis.html) was used to study the protein expression and promoter methylation levels of CDK12 in different cancer types and their adjacent tissues. Student’s t test was used to assess the significance of differences, with p < 0.05 regarded as statistically significant.

### Analysis of diagnostic value of CDK12

We explored the relationship between CDK12 expression and TNM staging in the TCGA clinical data. GraphPad Prism 9 was used for data visualization, and statistical analysis was performed using Student’s t test. Subsequently, the “timeROC” R package was used to perform ROC curve analysis based on sensitivity and specificity for the diagnostic accuracy of CDK12. The area under the curve (AUC) ranged from 1.0 (perfect diagnosis) to 0.5 (no diagnostic value)^43^.

### Analysis of the prognostic value of CDK12

To explore the correlation between CDK12 and prognosis, we extracted survival data from the TCGA database and used overall survival (OS), progression-free survival (PFS), and disease-specific survival (DSS) as indicators. For survival analysis, the Kaplan‒Meier method and log-rank test were used in each type of cancer. The survival curves were drawn using the “survival”, “surminer”, “limma”, and “ggpubr” R packages. Furthermore, the relationship between CDK12 and pancancer survival was plotted using the “forestplot” R package, and single-variable Cox regression was used to calculate the hazard ratio (HR) and p value.

### Pancancer analysis of the association between TMB or MSI and CDK12 gene expression

The correlation between CDK12 expression and TMB or MSI was elucidated by the Spearman correlation coefficient, and the TMB or MSI scores were obtained from TCGA pancancer mutation data. In the figure, the X-axis indicates the correlation coefficient of CDK12 with TMB or MSI, the Y-axis indicates different cancer types, the size of the circle represents the size of the correlation coefficient, and the different colours indicates the significance of the p value. The radar chart was obtained using the “ggstatsplot” R package. Cancer types with a statistically significant correlation (p < 0.05) between CDK12 expression and TMB or MSI are listed.

### Pancancer analysis of the relationship between CDK12 expression and tumour cell immune infiltration and immune checkpoint genes

To perform a reliable immunoreactivity assessment, we used “immunedeconv”, an R package that integrates six novel algorithms, including TIMER, xCell, MCP-counter, CIBERSORT, EPIC and quanTIseq, representing gene expression levels as log2 TPM values. With TIMER^44^and xCell^45^algorithms as the first choice, we generated multiple Spearman correlation heatmaps of immunoreactivity scores and the CDK12 gene based on a larger variety of immune cell types in different cancer types. SIGLEC15, IDO1, CD274, HAVCR2, PDCD1, CTLA4, LAG3 and PDCD1LG2 were the transcripts related to the immune checkpoint, and we extracted the expression levels of these 8 genes to observe the expression of immune checkpoint-related genes. The correlation between CDK12 and the immune checkpoint genes was plotted using the “reshape2” and “RColorBrewer” R packages. The X-axis in the figure represents different types of cancer, the Y-axis represents different immunoreactivity scores, and different colours represent the correlation coefficient. All statistical analyses were conducted with R software v4.0.3 (*p < 0.05, **p < 0.01, ***p < 0.001).

### Genomic alterations and the gene‒gene network of CDK12

The cBioPortal platform (http://www.cbioportal.org/) contains all tumour gene data from the TCGA database and is capable of providing multidimensional visualized data. We selected data from 30 cancers, with a total of 10,967 samples, and conducted further analysis within cBioPortal. Gene mutation and mutation loci information of CDK12 were obtained through the OncoPrint, Cancer Type Summary and Mutation modules. The Cancer Types Summary presents the mutation rate of target genes in various types of cancer in the form of bar graphs. OncoPrint presented the mutation, copy number, and expression of the target genes in all samples in the form of heatmaps. In addition, mutations were used to analyse the mutation types of CDK12 and the gene types significantly affected by the mutation.

The GeneMANIA database (http://www.genemania.org) is a user-friendly website that can find functionally similar genes based on a given gene list according to rich genome and proteome data. Through the platform for gene functional similarity detection in this database, genes similar to the CDK12 expression pattern were identified.

### Interaction between CDK 12 and chemical substances

The CTD database (http://ctdbase.org/) serves as a digital resource that is beneficial for elucidating the effects of chemicals on genes and uncovering new correlations in the molecular mechanisms^46^. We used this database to query for chemicals interacting with CDK12, and based on the chemicals that have similar interactions, we conducted an exploration of genes that were highly similar to CDK12.

### Supplementary Information

Supplementary Tables.

## Data Availability

Data are contained within the article.
