# Supplementary material for: CDK12 is a potential biomarker for diagnosis, prognosis and immunomodulation in pan-cancer
Source: Sci Rep. 2024 Mar 19;14:6574. doi: 10.1038/s41598-024-56831-7 (PMC10951204; doi:10.1038/s41598-024-56831-7)

| Type | **Pixel Count** | **Percentage contribution of High Positive** | **Percentage contribution of Positive** | **Percentage contribution of Low Positive** | **Percentage contribution of Negative** | **Score** |
| --- | --- | --- | --- | --- | --- | --- |
| Breast | 2940 | 4.5132 | 0 | 0 | 95.4868 | Negative |
| Breast cancer | 2940 | 3.7931 | 0.0702 | 62.3503 | 33.7864 | Low positive |
| Cervix | 2989 | 3.6886 | 0 | 0.5415 | 95.7699 | Negative |
| Cervical cancer | 2940 | 3.6797 | 0.1704 | 42.9983 | 53.1516 | Low positive |
| Colon | 3008 | 3.6417 | 0.9843 | 24.7375 | 70.6365 | Negative |
| Colorectal cancer | 3072 | 4.9339 | 7.533 | 43.5683 | 43.9648 | Low positive |
| Rectum | 3008 | 3.6569 | 0.9785 | 16.4271 | 78.9375 | Negative |
| Colorectal cancer | 3072 | 3.618 | 5.1499 | 31.9753 | 59.2568 | Low positive |
| Kidney | 3008 | 3.6913 | 0 | 18.7919 | 77.5168 | Negative |
| Renal cancer | 3072 | 3.6133 | 0.2279 | 45.1172 | 51.0416 | Low positive |
| Liver | 3008 | 3.6569 | 0 | 0 | 96.3431 | Negative |
| Liver cancer | 3072 | 3.6145 | 8.8245 | 64.2462 | 23.3148 | Low positive |
| Lung | 3055 | 5.836 | 0 | 22.0294 | 72.1346 | Negative |
| Lung cancer | 3120 | 3.5909 | 0.03212 | 35.7486 | 60.6284 | Low positive |
| Lymph node | 3008 | 3.6569 | 27.1609 | 58.9495 | 10.2327 | Negative |
| Lymphoma | 3072 | 3.6133 | 72.7865 | 23.6002 | 0 | Positive |
| Pancreas | 4526 | 2.9607 | 1.3699 | 52.8988 | 42.7706 | Negative |
| Pancreatic cancer | 4464 | 3.0633 | 11.2094 | 48.9222 | 36.8051 | Low positive |
| Prostate | 4526 | 3.4697 | 5.593 | 56.4578 | 34.4795 | Negative |
| Prostate cancer | 4599 | 2.9683 | 1.1873 | 47.4714 | 48.373 | Low positive |
| Stomach | 4536 | 2.9594 | 2.1422 | 35.9629 | 58.9355 | Negative |
| Stomach cancer | 4599 | 2.9354 | 9.002 | 71.2981 | 16.7645 | Low positive |
| Testis | 4599 | 3.2452 | 0.1202 | 19.7596 | 76.875 | Negative |
| Testis cancer | 4599 | 3.0689 | 3.4553 | 66.697 | 26.7788 | Low positive |
| Skin | 4526 | 3.6148 | 0.2158 | 54.5454 | 41.624 | Low positive |
| Skin cancer | 4599 | 2.9361 | 0.5002 | 27.0987 | 69.465 | Negative |
| Endometrium | 3008 | 3.6569 | 0.06649 | 42.65281 | 53.6238 | Low positive |
| Endometrial cancer | 3072 | 3.6537 | 0 | 27.8275 | 68.5188 | Negative |
| Thyroid | 4599 | 2.9367 | 0.5221 | 35.4579 | 61.0833 | Low positive |
| Thyroid cancer | 4599 | 3.3616 | 0 | 25.1743 | 71.4641 | Negative |
| Ovary | 4599 | 2.9354 | 2.9572 | 21.9265 | 72.1809 | Negative |
| Ovarian cancer | 4599 | 2.9521 | 2.2961 | 37.5705 | 57.1813 | Negative |
| Urinary bladder | 15129 | 1.6884 | 1.2183 | 13.2093 | 83.884 | Negative |
| Urothelial cancer | 15129 | 1.8152 | 3.8896 | 17.9068 | 76.3884 | Negative |

**Table S1**

**Table S2**

| Figure 1A | Sample size(normal) |
| --- | --- |
| Brain | 2642 |
| Pancreas | 332 |
| Bile Duct | 9 |
| Liver | 276 |
| Kidney | 121 |
| Lymph Gland | 929 |
| Stomach | 391 |
| Adrenal Gland | 258 |
| Colon | 820 |
| Rectum | 789 |
| Esophagus | 1456 |
| Prostate | 297 |
| Breast | 572 |
| Bladder | 40 |
| Lung | 637 |
| Uterus | 177 |
| Thyroid | 712 |
| Skin | 1810 |
| Cervix Uteri | 22 |
| Ovary | 180 |
| Thymus | 2 |
| Testis | 361 |

| Figure 1B | Sample size |
| --- | --- |
| Vulva/Vagina | 2 |
| Testis | 2 |
| Cervix | 19 |
| Head and Neck | 58 |
| Eye | 18 |
| Biliary Tract | 38 |
| Adrenal Gland | 1 |
| Ovary/Fallopian Tube | 65 |
| Soft Tissue | 45 |
| Fibroblast | 38 |
| Kidney | 44 |
| Thyroid | 16 |
| Prostate | 12 |
| Bone | 38 |
| Skin | 87 |
| Peripheral Nervous System | 36 |
| CNS/Brain | 98 |
| Pancreas | 53 |
| Uterus | 40 |
| Bowel | 74 |
| Bladder/Urinary Tract | 37 |
| Liver | 25 |
| Myeloid | 62 |
| Breast | 63 |
| Esophagus/Stomach | 71 |
| Pleura | 21 |
| Lymphoid | 156 |
| Ampulla of Vater | 4 |
| Lung | 185 |

| Figure 1 C | Sample size(cancer) |
| --- | --- |
| KICH | 65 |
| ACC | 79 |
| PCPG | 181 |
| LIHC | 371 |
| GBM | 153 |
| OV | 376 |
| KIRP | 290 |
| LGG | 513 |
| UVM | 80 |
| UCEC | 545 |
| THCA | 512 |
| KIRC | 532 |
| SARC | 260 |
| MESO | 87 |
| PAAD | 179 |
| THYM | 120 |
| BLCA | 406 |
| UCS | 57 |
| PRAD | 498 |
| CESC | 306 |
| DLBC | 48 |
| CHOL | 35 |
| SKCM | 471 |
| HNSC | 504 |
| LUAD | 516 |
| READ | 165 |
| COAD | 455 |
| LUSC | 501 |
| STAD | 375 |
| TGCT | 134 |
| BRCA | 1101 |
| ESCA | 163 |
| LAML | 150 |

| Figure 1 D/6/7/8/9/10/11 | Sample size |
| --- | --- |
| Normal | 7056 |
| Cancer | 3172 |

| Figure 4 | N | I | II | III | IV |
| --- | --- | --- | --- | --- | --- |
| BRCA | 114 | 183 | 615 | 247 | 20 |
| CHOL | 9 | 10 | 9 | 1 | 7 |
| COAD | 41 | 45 | 110 | 80 | 39 |
| ESCA | 11 | 13 | 78 | 55 | 9 |
| HNSC | 44 | 27 | 71 | 81 | 264 |
| KICH | 25 | 20 | 25 | 14 | 6 |
| KIRC | 72 | 267 | 57 | 123 | 84 |
| LIHC | 50 | 168 | 84 | 82 | 6 |
| LUAD | 59 | 277 | 125 | 85 | 28 |
| LUSC | 52 | 243 | 157 | 85 | 7 |
| STAD | 34 | 18 | 123 | 169 | 41 |
| THCA | 59 | 284 | 52 | 112 | 55 |

| Figure 12 CDK12 mRNA expression | Sample size |
| --- | --- |
| Deep Deletion | 12 |
| Shallow Deletion | 1428 |
| Diploid | 6407 |
| Gain | 1771 |
| Amplification | 270 |


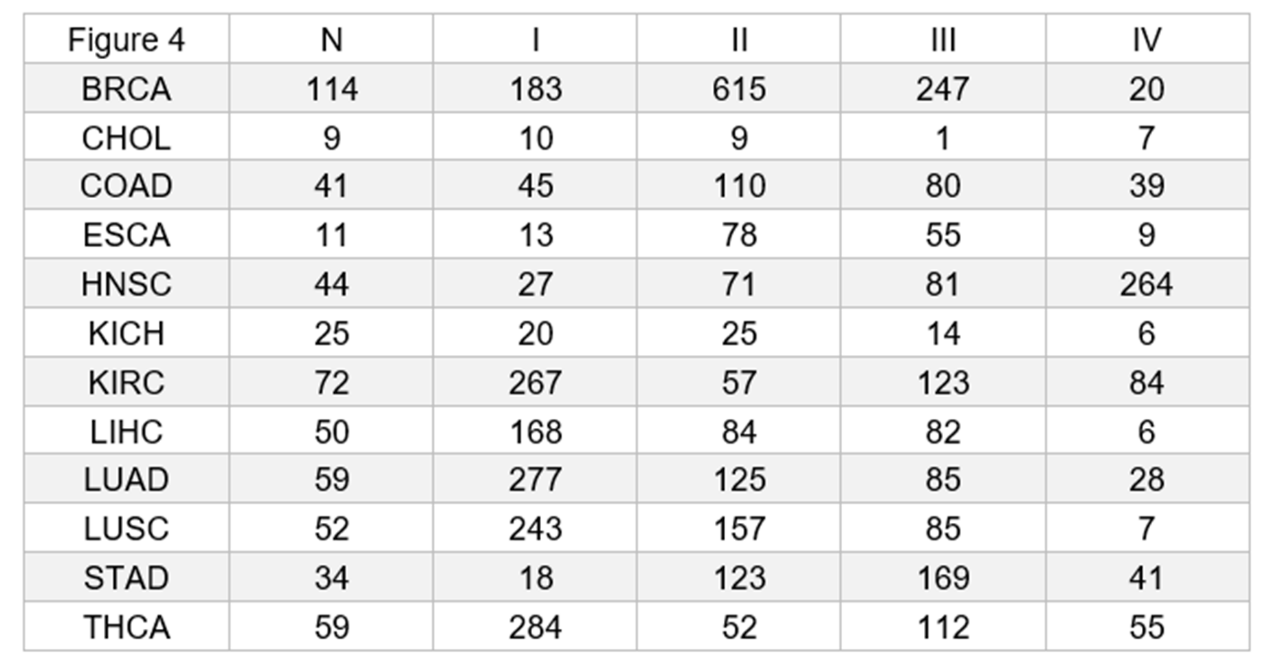

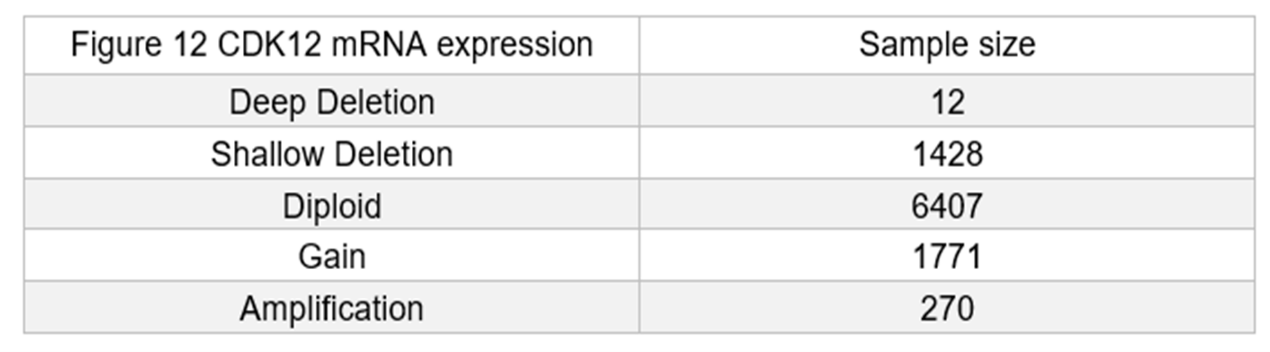


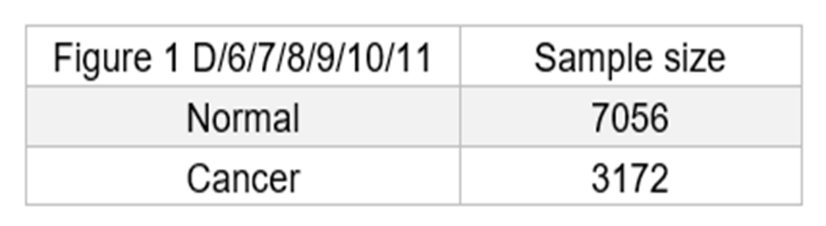


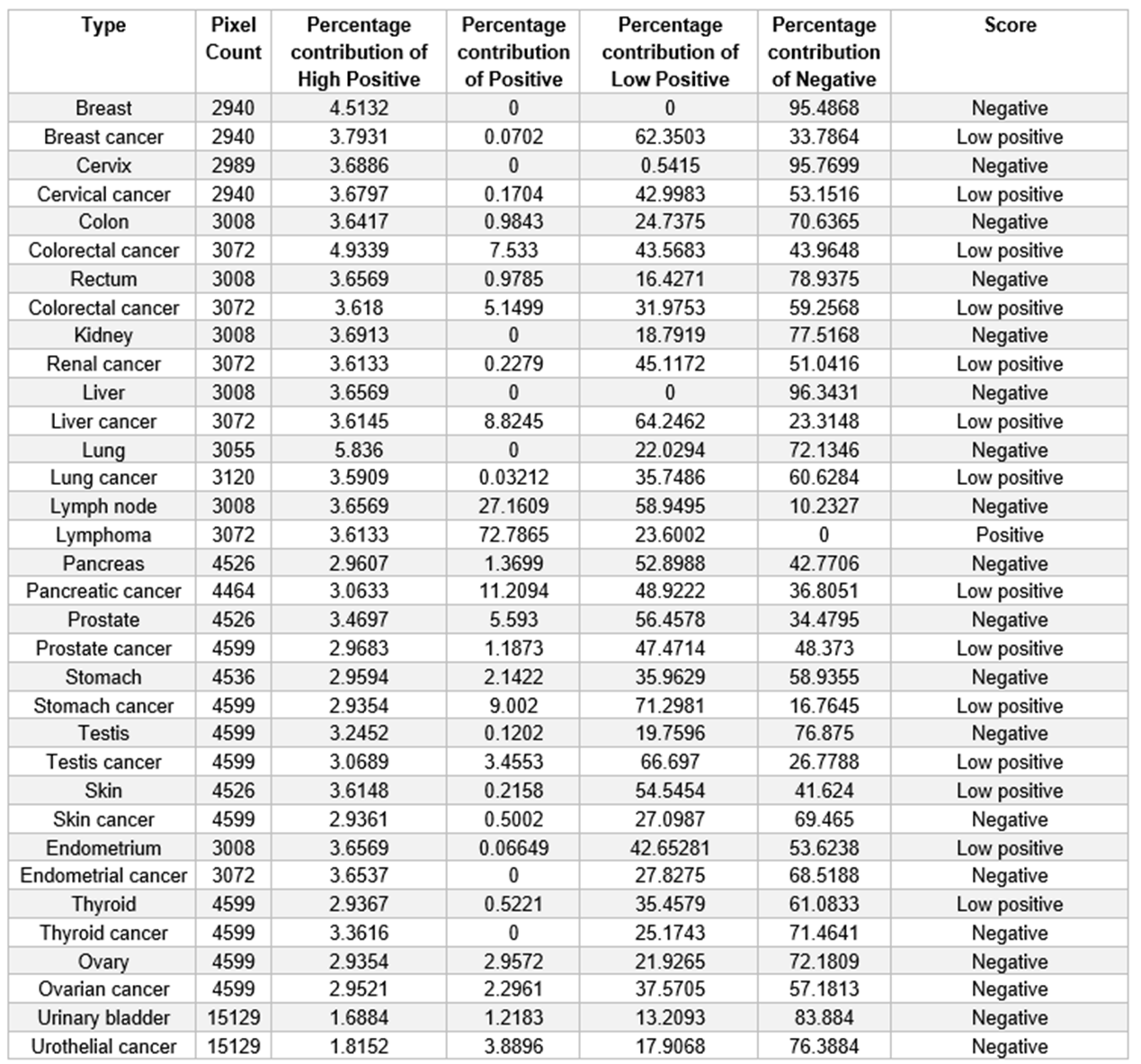

Supplement: Supplementary file 1 — Supplementary Tables. [file 41598_2024_56831_MOESM1_ESM.docx]
